# Determination of Homoarginine, Arginine, NMMA, ADMA, and SDMA in Biological Samples by HPLC-ESI-Mass Spectrometry

**DOI:** 10.3390/ijms141020131

**Published:** 2013-10-09

**Authors:** Luigi Servillo, Alfonso Giovane, Nunzia D’Onofrio, Rosario Casale, Domenico Cautela, Domenico Castaldo, Maria Luisa Balestrieri

**Affiliations:** 1 Department of Biochemistry, Biophysics and General Pathology, Second University of Naples, 80138 Naples, Italy; E-Mails: luigi.servillo@unina2.it (L.S.); alfonso.giovane@unina2.it (A.G.); nunzia.donofrio@unina2.it (N.D.); rosario.casale@unina2.it (R.C.); 2 Stazione Sperimentale per le Industrie delle Essenze e dei derivati dagli Agrumi (SSEA), Azienda Speciale della Camera di Commercio di Reggio Calabria, 89125 Reggio Calabria, Italy; E-Mails: dcautela@ssea.it (D.Cau.); dcastaldo@ssea.it (D.Cas.)

**Keywords:** homoarginine, arginine, NMMA, ADMA, SDMA

## Abstract

*N*^G^,*N*^G^-dimethyl-l-arginine (ADMA) and *N*^G^-methyl-l-arginine (NMMA) are endogenous inhibitors of nitric oxide synthase (NOS). In contrast, *N*^G^,*N*′^G^-dimethyl-Larginine (SDMA) possesses only a weak inhibitory potency towards neuronal NOS and it is known to limit nitric oxide (NO) production by competing with l-arginine for cellular uptake. The inhibition of NOS is associated with endothelial dysfunction in cardiovascular diseases as well in chronic renal failure. l-Homoarginine (HArg), a structural analog of l-arginine (Arg), is an alternative but less efficient substrate for NOS. Besides, it inhibits arginase, leading to an increased availability of l-arginine for NOS to produce NO. However, its relation with cardiovascular disease remains unclear. To date, several analytical methods for the quantitative determination of Arg, HArg, NMMA, AMDA, and SDMA in biological samples have been described. Here, we present a simple, fast, and accurate HPLC-ESI-MS/MS method which allows both the simultaneous determination and quantification of these compounds without needing derivatization, and the possibility to easily modulate the chromatographic separation between HArg and NMMA (or between SDMA and ADMA). Data on biological samples revealed the feasibility of the method, the minimal sample preparation, and the fast run time which make this method very suitable and accurate for analysis in the basic and clinical settings.

## 1. Introduction

The methylarginines, *N*^G^-methyl-l-arginine (monomethylarginine; NMMA), *N*^G^,*N*^G^-dimethyl-Larginine (asymmetric dimethylarginine, ADMA), and *N*^G^,*N*′^G^-dimethyl-l-arginine (symmetric dimethylarginine; SDMA) as well as l-arginine (Arg) and l-homoarginine (HArg) are important players of the nitric oxide (NO) metabolism [1]. NO, an important regulator of vascular homeostasis, is synthesized from l-arginine by NO synthase (NOS) [2,3]. The reduction of its synthesis promotes endothelial dysfunction and correlates with risk factors for cardiovascular disease (CVD) [2,3]. Circulating ADMA and NMMA are inhibitors of NOS activity [1,4,5]. SDMA, considered not to inhibit NOS, has been shown to possess a weak inhibitory potency towards neuronal NOS [6].

Free ADMA, SDMA, and NMMA are supposed to be produced in the human body through the methylation of protein arginine residues by protein arginine methyltransferases (PRMT) and released during proteolysis of the methylated proteins [4]. To maintain a steady pool size, free methylarginines are metabolized by dimethylarginine dimethylaminohydrolase (DDAH) enzymes. An imbalance in this pool, due to PRMT or DDAH dysfunction, might increase cardiovascular risk. Indeed, elevated levels of ADMA, which is considered a risk factor for CVD, have been detected in a large number of diseases associated with an impaired endothelial l-arginine-NO pathway, such as atherosclerosis, hypertension, hypercholesterolemia, chronic heart failure, type 2 diabetes mellitus, stroke, and hyperhomocysteinemia [7–10].

Notably, we recently showed that these three methylarginines, in addition to being produced endogenously, can also be taken daily in conspicuous amounts through the diet [11]. Indeed, ADMA, SDMA, and NMMA are ubiquitous in vegetables which represent an important part of the human daily diet. Among vegetables, soybean, rye, sweet pepper, broad bean, and potato contain considerable amounts of ADMA, SDMA, and NMMA [11]. The highest mean content of NMMA has been detected in sweet pepper and potato (7.6 and 7.2 μmoles/kg, respectively) which also contain about 9.6 and 4.1 μmoles/kg of ADMA and 0.8 and 0.6 μmoles/kg of SDMA, respectively.

Homoarginine (HArg) interferes with the regular NO production as it competes with arginine for the NO synthesis although it is a less efficient NOS substrate than arginine [12]. However, studies on the relationship between HArg levels and CVD had seemingly contradicting outcomes. Indeed, both high and low HArg levels have been linked to cardiovascular detrimental effects. High HArg levels have been found to induce increased vascular resistance, whereas prospective studies showed that low HArg levels were associated with cardiovascular mortality and stroke [13–15].

The development of rapid and feasible methods for the combined determination of Arg and its metabolites and analogs is still challenging as it might have an impact on NO metabolic studies. Actually, several methods for the determination of Arg and related metabolites in biological fluids, usually associated with numerous difficulties due to the extraction and derivatization steps, are available [16–20]. We recently reported an original chromatographic procedure that can be employed to analyze ADMA, SDMA, and NMMA with ESI-mass spectrometry detection without needing derivatization [11]. This method, performed with a short silica column (Supelcosil^™^ LC-Si 3.3 cm × 4.6 mm i.d., 3 μm particle size) utilizes isocratic elution conditions which represent a noticeable advantage in terms of baseline stability during the chromatography with MS detection and spare time for skipping column re-equilibration after each analysis [11]. The use of the Supelcosil^™^ LC-Si column, usually employed with normal phase elution mode, allows the separation of NMMA, SDMA, and ADMA using a purely aqueous mobile phase and with retention times of 7, 9, and 10.5 min, respectively. However, the elution conditions utilized in that work [11] although allowing short analysis time result in poor resolution between HArg and NMMA, which was unimportant in the particular case as HArg was absent in the vegetal samples analyzed in that research.

Taking into account the emerging role of HArg as a risk factor in CVD, there is increasing interest in measuring HArg levels in human subjects and cell culture. Here, we report a novel and feasible application of the HPLC-ESI-mass spectrometry detection using the Supelcosil^™^ LC-Si column which allows an accurate and combined determination of NMMA, SDMA, ADMA, Arg, and HArg in biological fluids such as plasma and urine.

## 2. Results and Discussion

The chromatography was performed with a short silica column (Supelcosil^™^ LC-Si 3.3 cm × 4.6 mm i.d., 3 μm particle size), employing isocratic elution conditions. This procedure, proven to be suitable for polar substance analysis with ESI-MS/MS detection (Table 1), does not require sample derivatization. Importantly, the minimal sample preparation, *i.e*., resuspension in 0.1% solution of formic acid in water after protein precipitation makes this step inexpensive and no time-consuming. The effect of the deproteinization step was evaluated by adding NMMA as internal standard in some samples in which this substance was found absent. The recoveries resulted in the range of 94%–98%. In order to assess linearity, five aqueous solutions of the standards at various concentrations were prepared as reported in the experimental section. The correlation coefficients (*R*^2^) of the calibration curves resulted in the range 0.988–0.998. The limit of quantification in plasma (LOQ), which was practically the same for all the five substances, was 0.06 μM. The linear range and the LOQ are similar to previously described methods.

**Figure 1:**
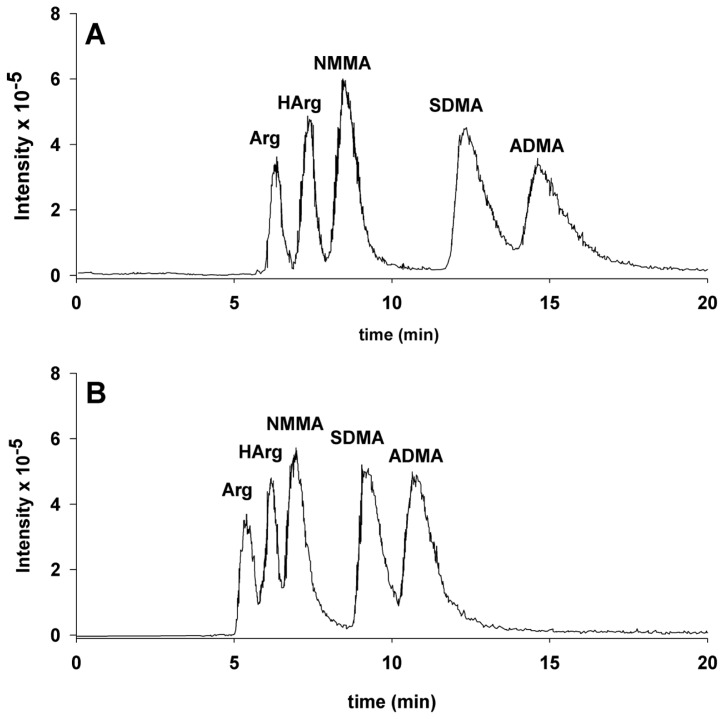
Chromatographic separation of Arg, HArg, NMMA, SDMA, ADMA with full-scan detection. (**A**) Elution pattern of the chromatography performed utilizing 10% of Sol. B (100 mM ammonium formate in water titrated to pH 4.5 with formic acid) and 90% Sol. A (0.1% formic acid in water); (**B**) Elution pattern of the chromatography performed utilizing 20% of Sol. B and 80% Sol. A. The concentrations were 5.1 μM for Arg, 5.9 μM for HArg, 7.4 μM for NMMA, 8.6 μM for SDMA and 8.4 μM for ADMA. The retention times for the chromatography in **panel A** resulted 6.3 min, 7.4 min, 8.4 min, 12.3 min and 14.6 min for Arg, HArg, NMMA, SDMA and ADMA, respectively. The retention times for the chromatography in **panel B** resulted 5.4 min, 6.1 min, 7.0 min, 9.1 min and 10.7 min for Arg, HArg, NMMA, SDMA and ADMA, respectively.

The chromatographic separation of Arg, HArg, NMMA, SDMA, and ADMA is shown in Figure 1.

Perhaps the most important feature of the reported chromatographic analyses is the noticeable dependence on the content of ammonium formate in the elution solvent of the retention times and, hence, of the resolution of those substances. In fact, when the chromatography is performed utilizing an eluent of 0.1% solution of formic acid in water (Sol. A), SDMA and ADMA elute at high retention times (above 60 min) as very broad peaks. Instead, the effect of ammonium formate addition to the eluent is clearly shown by the comparison of the two panels shown in Figure 1.

In panel A, which reports the elution pattern of the chromatography performed utilizing 10% of Sol. B and 90% Sol. A, an almost complete resolution among Arg, HArg and NMMA can be observed. In contrast, in panel B, which reports the chromatography performed utilizing an eluent composition of 20% Sol. B and 80% Sol. A, the peaks of HArg and NMMA are more poorly resolved. It is important to note that the MS/MS fragmentation pattern of NMMA and HArg are almost identical. Indeed, it is possible to quantify by MS/MS mass spectrometry NMMA also in the presence of HArg as NMMA shows an intense fragment at *m*/*z* 74 that is absent in the MS/MS fragmentation pattern of HArg. The converse does not hold as all the analytically important MS/MS fragments of HArg are common with NMMA. For this reason if one wants to quantify by mass spectrometry HArg in presence of NMMA, it is mandatory to obtain the chromatographic separation of the two substances and this can be obtained utilizing the chromatographic condition of panel A. Indeed, we found that a reasonably good separation can be obtained with molar ratios of HArg/NMMA up to 10:1.

When determining the levels of Arg and its metabolites in samples of plasma from human healthy volunteers (*n* = 12), the results indicated that the concentrations we found were comparable to the mean values previously reported [16–20]. Specifically, the plasma mean concentrations detected with the method here described were 86 ± 12.5 μM for Arg, 2.3 ± 0.74 μM for HArg, 0.67 ± 0.04 μM for ADMA, 0.522 ± 0.08 μM for SDMA, and 0.11 ± 0.09 μM for NMMA. The urine mean concentrations were 12.1 ± 1.5 μM for Arg, 2.1 ± 0.31 μM for HArg, 45.2 ± 13.5 μM for ADMA, and 48.4 ± 12.1 μM for SDMA. The data reported are expressed as mean ± standard deviation (*n* = 12).

## 3. Experimental Section

### 3.1. Reagents

ADMA (*N*^G^,*N*^G^-dimethyl-l-arginine dihydrochloride), SDMA (*N*^G^,*N*′^G^-dimethyl-l-arginine diphydroxyazobenzene-p′-sulfonate salt, NMMA (*N*^G^-methyl-l-arginine acetate salt), homoarginine, formic acid, ammonium formate and the 0.1% solution of formic acid in water used for the LC–ESI–MS analyses were from Sigma–Aldrich (Milan, Italy).

### 3.2. Sample Preparation

Plasma and urine samples were obtained from human healthy subjects. An aliquot (100 μL) was deproteinized by adding 300 μL of pure methanol. Samples were mixed for 10 s, stored at −20 °C for 1 h, and then centrifuged at 10,000 × *g* for 10 min at 4 °C. Supernatants were recovered, evaporated and then dissolved in 100 μL of 0.1% solution of formic acid in water. An aliquot of 20 μL was used for HPLC-ESI-MS/MS analyses.

### 3.3. HPLC–ESI–MS/MS Instrumental Conditions

The HPLC-ESI-MS analyses were performed with some modification to the previously described method [11]. Briefly, analyses were performed with an HPLC Agilent 1100 series equipped with on line degasser and automatic injector coupled on-line with an Agilent LC-MSD SL quadrupole ion trap as described [11]. The measurements were performed from the peak area of the Extracted Ion Chromatogram (EIC). The quantification was achieved by comparison with the calibration curves obtained with standard solutions prepared at a concentration of 2000 mg/L. Additional calibration levels (25, 5, 2, 1 and 0.1 mg/L) were prepared by serial dilution with water containing 0.1% formic acid and stored at 4 °C. The mass cut-off and the fragmentation amplitude were optimized in order to obtain the most efficient MS/MS transitions from the positively charged precursor ion [M + H^+^] to the fragment ions. Multiple reaction monitoring was used for analyte quantification, the MS/MS transitions utilized were 175.1→116 for Arg, 189.2→144 for HArg, 189.2→74 for NMMA, 203.2→172 for SDMA, 203.2→158 for ADMA. Successively, volumes of 10–20 μL of standard solutions or samples were analyzed by HPLC–ESI–MS/MS by using the silica column Supelcosil^™^ LC-Si 3.3 cm × 4.6 mm i.d., 3 μm particle size. The elution was performed isocratically at a flow rate of 100 μL/min with an eluent obtained by mixing in the due ratio a solution of 0.1% formic acid in water (Sol. A) and 100 mM ammonium formate in water titrated to pH 4.5 with formic acid (Sol. B). The retention times and peak areas of the monitored fragment ions were determined by the Agilent software Chemstation version 4.2.

## 4. Conclusions

Attractive features of the HPLC–ESI-MS/MS method described here for the determination of Arg, HArg, NMMA, ADMA, and SDMA in biological samples are: (i) the minimal sample preparation without the need of derivatization; (ii) the modulation of the analysis time by suitably modifying the content of ammonium formate in the eluent and, most of all; (iii) the feasibility which offers the opportunity to modulate, depending on the specific experimental requirements, the chromatographic separation between HArg and NMMA (or between SDMA and ADMA).

## Figures and Tables

**Table 1:** Mass spectrometric conditions. The HPLC Agilent 1100 series was equipped with on line degasser and automatic injector coupled on-line with an Agilent 1100 LC/MSD SL quadrupole ion trap.

Agilent LC–MSD SL quadrupole ion trap settings	
MS acquisition	ESI in positive ion mode
Nebulizer pressure	30 psi
Drying temperature	350 °C
Drying gas	7 L/min

	Ion Charge Control (ICC), target set at 30,000, maximum accumulation time at 20 ms
